# Residual antibacterial effects of a mixture of silver nanoparticles/calcium hydroxide and other root canal medicaments against *Enterococcus faecalis*

**DOI:** 10.1016/j.jds.2021.11.013

**Published:** 2021-12-07

**Authors:** Farzaneh Afkhami, Golriz Rostami, Sharareh Batebi, Abbas Bahador

**Affiliations:** a Department of Endodontics, School of Dentistry, Tehran University of Medical Sciences, International Campus, Tehran, Iran; b Laser Research Center of Dentistry, Dentistry Research Institute, Tehran University of Medical Sciences, Tehran, Iran; c General Dentist, Tehran, Iran; d Oral Microbiology Laboratory, Department of Microbiology, School of Medicine, Tehran University of Medical Sciences, Tehran, Iran

**Keywords:** Calcium hydroxide, Chlorhexidine, Double antibiotic paste, Nanoparticles, Root canal medicament, Residual antibacterial effect

## Abstract

**Background/purpose:**

Intracanal medicaments with high residual inhibition effects are used to achieve successful endodontic treatment and prevent reinfection. This in vitro study aimed to assess antimicrobial residual effects of different intracanal medicaments against *Enterococcus faecalis (E. faecalis)* in root dentin one week after drug therapy.

**Materials and methods:**

Seventy human teeth were standardized to have 15 mm of length and were prepared by ProTaper rotary system. The teeth were randomly divided into six experimental (n = 10) and two control (n = 5) groups and subjected to drug therapy as follows; group CHX: 2% chlorhexidine gel, group CH: calcium hydroxide paste, group CH/CHX: mixture of calcium hydroxide and 2% CHX, group TAP: triple antibiotic paste, group DAP: double antibiotic paste, group CH/AgNPs: mixture of CH paste and silver nanoparticle suspension. After one week, the medicaments were removed from the root canals and *E. faecalis* was inoculated into the canals. The teeth were incubated at 37 °C for 24 h. Using a size 40 hedstrom file, dentin chips were collected and number of colony forming units were counted.

**Results:**

The difference between all experimental groups was statistically significant in this regard (P < 0.05) except for DAP and TAP groups, in which, no bacterial colony was noted after culture. After DAP and TAP, the lowest colony count was noted in CH/AgNPs, CH/CHX and CHX groups. The highest colony count was noted in CH group.

**Conclusion:**

Our results showed favorable residual antibacterial effects of TAP, DAP and CH/AgNP medicaments after one week of application.

## Introduction

Residual microorganisms in the root canal system, their recolonization and metabolic products as well as coronal leakage are the main causes of endodontic treatment failure.^1^^,^^2^ The main goal of endodontic treatment is to eliminate the microorganisms from the root canal system and prevent reinfection.^3^^,^^4^ Mechanical instrumentation, use of irrigating solutions and intracanal medicaments^3^ are among the suggested techniques for this purpose. Re-infection of the root canal system may occur due to regrowth of residual microorganisms.^5^

In infected canals, intracanal medicaments are used for different purposes such as complete elimination of residual bacteria after instrumentation, decrease inflammation in pulp and periapical tissues, or to serve as a barrier against coronal leakage of temporary filling materials.^6^ Calcium hydroxide (CH) is among the most commonly used intracanal medicaments.^4^^,^^7^ Its advantages include wide spectrum antimicrobial activity, biocompatibility and alkaline pH.^8^ Moreover, CH can prevent reinfection of the root canal system and impair nutritional supply to residual microorganisms in the root canal system.^7^ Due to the complexity of the root canal system and the fact that several microorganisms are often involved in root canal infections, one antibiotic cannot sterilize the entire root canal system and a combination of several antibiotics is required to eliminate this complex flora.^9^ A combination of three antibiotics namely metronidazole, ciprofloxacin and minocycline known as triple antibiotic paste (TAP) was suggested for disinfection of root canal system.^10^^,^^11^ Minocycline in TAP causes significant coronal discoloration. Thus, it was eliminated from the formulation of double antibiotic paste (DAP), which has metronidazole and ciprofloxacin.^12^

Silver nanoparticles (AgNPs) have high antibacterial activity because of their small particles and high surface area.^10^^,^^13^ Although CH is among the most commonly used disinfectants of the root canal system, it cannot effectively eliminate *Enterococcus faecalis (E. faecalis)* from the root canal system. Thus, studies have recommended to use a combination of CH with other antimicrobial agents such as AgNPs. Evidences show that combination of these two intracanal medicaments significantly improves antibacterial activity against *E. faecalis* without evident side effects (e.g. discoloration).^14^^,^^15^

A successful endodontic treatment requires the use of antimicrobial agents with high substantivity. Substantivity is defined as durability of antimicrobial effects of irrigating solutions and intracanal medicaments over time.^16^ Substantivity refers to long contact between a material such as chlorhexidine (CHX) and a surface (such as oral mucosa, dental plaque and tooth surface); this interaction can be more complex than a simple deposition mechanism.^17^ This results in long term effect of drugs and medications.^18^ Optimal efficacy of CHX as an antimicrobial agent and intracanal medicament depends on its substantivity in dental structures. The substantivity of CHX depends on its bond to organic and inorganic components of dentin. Also, this property results in formation of a more stable layer, which increases the success of endodontic treatment.^19^ Antimicrobial substantivity of CHX depends on the number of molecules in contact with dentin. Thus, intracanal medicaments with higher concentration of CHX can increase resistance to bacterial colonization.^5^

In search of the literature, few studies have evaluated the residual antibacterial effects of medicaments. Thus, this in vitro study aimed to compare residual antibacterial effects of CHX, CH, CHX/CH, DAP, TAP and CH/AgNPs against *E. faecalis* growth in root canal one week after drug therapy.

## Materials and methods

The study protocol was approved in the ethics committee of Tehran University of Medical Sciences (approval no: REC.1395.252). Seventy single-rooted, sound human teeth with mature apices, and no cracks or resorption were chosen for this study. Parallel periapical radiographs were taken to ensure that the teeth had a single canal. Tooth crowns were cut vertical to the longitudinal axis of the tooth by high-speed handpiece and fissure bur (Dentsply International, York, PA) under water and air spray such that the remaining root length was 15 mm. A #15 K file (Dentsply Maillefer, Ballaigues, Switzerland) was introduced into the root canal until its tip was visible at the apex. The working length was determined 1 mm short of this length. Root canals were instrumented using ProTaper files (Dentsply Maillefer, Ballaigues, Switzerland). S1, S2, F1, F2, F3, F4 and F5 were used, respectively. RC-Prep was used (Premier, Norristown, PA, USA) as lubricant and 1 mL of 2.5% sodium hypochlorite was used for irrigation between files.

Smear layer was removed using 1 mL of 17% EDTA (Master-dent, Charlotte, NC, USA) and 1 mL of sodium hypochlorite each for 3 min. Then, 5 mL of sterile saline was used for final rinse. To prevent apical leakage, apex of all teeth was sealed with self-cure glass ionomer (GC Gold Label, Kyoto, Japan). The entire external root surface was coated with two layers of nail varnish except for the orifice to prevent microbial contamination from the outer surface. The teeth were then autoclave-sterilized at 121 °C for 30 min. To ensure sterilization, microbial culture of some samples was done randomly.

### Intracanal dressings

The teeth were randomly divided into six experimental groups (n = 10) and two control groups (n = 5) and treated as follows:

Group CHX: 2% CHX (Sigma–Aldrich, St Louis, MO, USA) gel.

Group CH: CH paste (Meta Biomed, Cheongju, Korea).

Group CH/CHX: Mixture of CH and 2% CHX gel in 1:1 ratio (w:w).

Group TAP: Metronidazole (Mast laboratories LTD, Merseyside, UK), ciprofloxacin (Mast laboratories LTD) and minocycline (Mast laboratories LTD); 0.5 mg of each antibiotic was mixed with 1 mL of saline.

Group DAP: Metronidazole and ciprofloxacin; 0.5 mg of each antibiotic was mixed with 1 mL of saline.

Group CH/AgNPs: CH and silver nanoparticle suspension (mean particle size of 30 nm with 100 ppm concentration) (Nanopoosheshfelez, Tehran, Iran) were mixed in 1:1 ratio (w:w).

Positive control group: Saline.

Negative control group: No drug therapy.

The medicaments were delivered into the canals using a size 40 sterile Lentulo (Mani Inc., Tochigi, Japan) with a low-speed handpiece under a hood (Nuaire, Plymouth, MN, USA) in sterile conditions. The teeth were incubated at 37 °C for one week and 100% moisture. Next, root canals were rinsed with 1 mL of sterile saline by a 30 gage needle 1 mm short of the working length. Root canal walls were then cleaned of residual medicaments using a #40 sterile K file and rinsed with 5 mL of sterile saline. Root canals were then dried using #40 sterile paper points (Meta Biomed, Cheongju, Korea).

### Bacterial culture

*E. faecalis* (ATCC 29212) was cultured in aerobic conditions in brain heart infusion (BHI) broth (Merck, Hamburg, Germany) at 37 °C. Bacterial suspension was prepared of bacteria in logarithmic growth phase containing 1.5 × 10^8^ colony forming units (CFUs)/mL using a spectrophotometer (Eppendorf, Hamburg, Germany) (optical density of 0.08–0.1 at 600 nm). This concentration was confirmed by culture. In aseptic conditions under a biological hood, 10 μL of this suspension was added to root canal of teeth in each group except for the negative control group and incubated at 37 °C for 24 h.

### Microbiological sampling

After completion of incubation, root canals in each group were rinsed with 5 mL of sterile saline and dried with #40 sterile paper points. Using a sterile #40 Hedstrom file (Mani Inc, Tochigi, Japan), 0.008 g of dentin chips were collected of each canal and separately placed in sterile microtubes.

To ensure absence of medicaments in dentin chips, 1 mL of sterile phosphate buffered saline (PBS) was added to each microtube containing dentin chips, and microtubes were centrifuged at 13,000 g at 4 °C for 5 min. After centrifugation, PBS was removed from microtubes and 1 mL of sterile PBS was added to each tube again. This process was repeated three times. In final rinse, after removing the PBS, 1 mL of BHI broth was added to each microtube containing dentin chips. After serial dilution in a 96-well plate, 10 μL of each dilution was spread-cultured on BHI agar. The plates were incubated in aerobic conditions at 37 °C for 24 h and colony counting was done using the technique described by Miles et al.^20^ The colony counts were reported in CFUs/mL.

### Statistical analysis

Data were analyzed using SPSS version 16.0 (SPSS Inc., Chicago, IL, USA). One-way ANOVA was used to compare the mean CFUs among the groups. P < 0.05 was considered statistically significant.

## Results

Table 1 shows the number of *E. faecalis* CFUs after 24 h of incubation in the study groups.

**Table 1:** Mean colony count (CFUs/mL) in the groups after drug therapy.

Group	N	Mean	Std. Deviation	Minimum	Maximum
CH	10	1.79 × 10⁵	2.40 × 10^4^	1.42 × 10⁵	2.13 × 10⁵
CHX	10	7.70 × 10^4^	1.31 × 10^4^	4.80 × 10^4^	9.20 × 10^4^
CH/CHX	10	3.82 × 10^4^	9.80 × 10^3^	2.20 × 10^4^	5.70 × 10^4^
CH/AgNps	10	2.85 × 10^3^	6.45 × 10^2^	1.90 × 10^3^	4.10 × 10^3^
TAP	10	0.00	0.00	0.00	0.00
DAP	10	0.00	0.00	0.00	0.00
Positive control	5	2.27 × 10⁵	4.07 × 10^4^	1.84 × 10⁵	2.95 × 10⁵
Negative control	5	0.00	0.00	0.00	0.00

CH: Calcium hydroxide, CHX: Chlorhexidine, CH/AgNPs: mixture of CH and silver nanoparticles, CH/CHX: Mixture of calcium hydroxide and CHX, TAP: Triple antibiotic paste, DAP: Double antibiotic paste.

Bacterial growth was noted in the positive control group while no growth was noted in the negative control group. No *E. faecalis* growth was noted in DAP and TAP groups. The difference between these two groups and other groups was significant (P < 0.05). The difference in this regard among other groups was also significant (P < 0.001) such that the least growth was noted in CH/AgNPs, CH/CHX and CHX groups, respectively. The CH group showed the highest colony count.

## Discussion

The results demonstrated a high residual antibacterial activity in DAP, TAP and combination of AgNPs with CH groups against *E. faecalis*.

Mechanical preparation and irrigation of root canal cannot completely eliminate all microorganisms from the infected root canal system and this may result in root canal failure. Using an adjunct method such as intracanal medicaments is suggested for disinfection of root canal system.^21^ Therefore, it may be stated that the main objective of intracanal medicaments is to eliminate the remaining microorganisms in the root canal system after chemomechanical instrumentation and prevent their reentry in-between treatment sessions.^22^

Microorganisms resistant to conventional root canal treatment are the main cause of endodontic failure. *E. faecalis* is the culprit in such cases. It is a facultative anaerobic gram-positive microorganism and its prevalence in secondary endodontic infection is nine times higher than that in primary endodontic infection.^23^ *E. faecalis* is among the most resistant microorganisms responsible for secondary root canal infection and can be a major cause of endodontic failure. Resistance of *E. faecalis* in the root canal system is due to its deep penetration into dentinal tubules, bond to dentin collagen, high resistance to environmental stresses, low sensitivity to antimicrobial agents and its ability to deactivate these agents.^21^^,^^24^ Due to the correlation of *E. faecalis* with root canal failure and its resistance to antibacterial agents, this study used *E. faecalis* for inoculation of root canal system.^25^

Most studies evaluated root canal contamination by taking samples from the root canal system using paper points. The limitation of this technique is that bacterial sampling is done only from the fluid inside the root canals.^26^ In our study, dentin chips were collected by using Hedstrom files in order to isolate bacteria penetrated deep into dentinal tubules.

Chlorhexidine is a wide-spectrum antibacterial agent with optimal substantivity. This unique property of CHX is related to the positive charge of CHX molecules, which can be absorbed into dentin and remain there for long periods of time and prevent bacterial colonization. Its antimicrobial substantivity depends on the number of CHX molecules exposed to dentin surface. Thus, using CHX with high concentration as intracanal medicament increases resistance to microbial colonization.^5^ Rosenthal et al. showed that antimicrobial activity of CHX in root canal can remain for more than 12 weeks.^27^ Mahendra et al. indicated that antimicrobial substantivity of CHX decreases over time and this property for 2% CHX is higher than that of other concentrations.^28^ Thus, 2% CHX concentration was used in our study.

Calcium hydroxide is another commonly used intracanal medicament. When CH is used alone, its antibacterial effect on *E. faecalis* is high in the first 24 h. But after 72 h, its effect on *E. faecalis* decreases. The reason is dilution of drug over time. However, when CHX is used alone, its antibacterial effect on *E. faecalis* increases in the first 24–72 h and remains high; this is due to the substantivity of this agent^.29^ In a study by Ballal et al., combination of CHX and CH yielded less anti-microbial effect than CHX alone. This can be due to difference in pH of the two drugs and attachment of CHX molecules to CH ions and inhibition of release of CHX molecules.^29^ Several studies have assessed the anti-microbial activity of CHX and CH. However, our study showed that this combination yielded enhanced residual antibacterial effect.

In order to confront complex root canal infection, a combination of antibiotics, instead of just one, is required. TAP is a combination of antibiotics with the greatest efficacy to decrease resistant bacterial strains in root canals, which includes metronidazole, ciprofloxacin and minocycline.^30^ There are two concerns with regard to the use of TAP: (1) Emergence of bacterial resistance and (2) tooth discoloration, which is due to the presence of minocycline in TAP and photo-initiated reaction of this antibiotic. Minocycline can bond to calcium ions present in dentin and create an insoluble complex, causing discoloration in dentin.^9^ Thus, DAP (metronidazole and ciprofloxacin) is used to prevent tooth discoloration.

Mohammadi et al. showed that the advantage of minocycline is its easy bond to dentin and its later release without losing its antibacterial activity. This creates a source of active antibacterial agent that has a sustained release from dentin surface.^30^ Sabrah et al. reported that tetracyclines such as minocycline can bond to dentin and collagen fibers present in root canal.^31^

TAP and DAP can induce long-term changes in dentin. These changes include change in physical structure of dentin or inactivation of growth factors. Considering these alterations, these antibiotic combinations can demineralize dentin matrix and increase fracture.^32^ Sabrah et al.^31^ reported that the antibacterial substantivity of DAP was longer than that of TAP. This indicates that components of DAP (metronidazole and ciprofloxacin) are more effective than minocycline present in TAP for bond to dentin. This can be due to low molecular weight of these two antibiotics, which enables them to penetrate deeper into dentin. Moreover, minocycline (compared to ciprofloxacin) has higher dissolution ability. Therefore, it may be stated that DAP is more resistant than TAP to elimination from root dentin, which results in greater antibacterial substantivity of DAP compared to TAP.^31^ In our study, drug therapy with DAP and TAP for one week resulted in no bacterial growth. However, Sabrah et al. reported bacterial growth after drug therapy with these agents.^31^ This controversy in the results may be due to difference in study samples. In their study, standardized dentin blocks were used while we used extracted teeth to better simulate the clinical setting. In this model, complete elimination of antibiotic from the root canal is more difficult and no bacterial growth in this group may be due to residual antibiotics in the root canal. Berkhoff et al. showed that different root canal irrigating solutions cannot completely eliminate these pastes from root dentin.^33^ Hohino et al. indicated that effective concentration of TAP is in the range of 0.003–1.5 mg/mL.^34^ In our study, 1.5 mg/mL concentration was used to ensure its efficacy against *E. faecalis*, which may suggest that no bacterial growth in these two experimental groups may be due to high concentration of these pastes.

Nanoparticles such as AgNPs have extensive antimicrobial activity and have low risk of emergence of microbial resistance.^35^ Antimicrobial efficacy of AgNPs against *E. faecalis* biofilm has been previously evaluated. Javidi et al. showed that combination of CH with AgNPs even after one day results in complete and efficient elimination of *E. faecalis*.^36^ The mechanism of activity of NPs is creation of cavity in cell wall of bacteria and decrease of bacterial adhesion and eventual prevention of bacterial biofilm formation.^36^ Also, they can decrease bacterial invasion to dentin.^37^ Afkhami et al. reported that AgNPs as carrier for CH for one week were the most effective for elimination of *E. faecalis* biofilm from dentin compared to other test groups.^15^ Thus, combination of CH and AgNPs not only enhances antibacterial activity, but also increases the residual antibacterial activity of CH as intracanal medicament.

Further researches should be conducted to evaluate the different intervals and concentrations of these medicaments to achieve optimal residual antibacterial activity.

This study showed that DAP, TAP and combination of AgNPs with CH had high residual antibacterial effect against *E. faecalis* and can be used as intracanal medicaments in-between root canal treatment sessions to achieve optimal prevention of bacterial regrowth.

## Funding

This study was supported by 10.13039/501100004484Tehran University of Medical Sciences (International Campus).

## Declaration of competing interest

The authors have no conflicts of interest relevant to this article.

## References

[1] Jain P., Yeluri R., Garg N. (2015). A comparative evaluation of the effectiveness of three different irrigating solution on microorganisms in the root canal: an *in vivo* study. J Clin Diagn Res.

[2] Bicego-Pereira E.C., Barbosa-Ribeiro M., de-Jesus-Soares A. (2020). Evaluation of the presence of microorganisms from root canal of teeth submitted to retreatment due to prosthetic reasons and without evidence of apical periodontitis. Clin Oral Invest.

[3] Komorowski R., Grad H., Wu X.Y., Friedman S. (2000). Antimicrobial substantivity of chlorhexidine-treated bovine root dentin. J Endod.

[4] Rahimi S., Janani M., Lotfi M. (2014). A review of antibacterial agents in endodontic treatment. Iran Endod J.

[5] Mohammadi Z., Abbott P.V. (2009). Antimicrobial substantivity of root canal irrigants and medicaments: a review. Aust Endod J.

[6] Chong B., Ford T.P. (1992). The role of intracanal medication in root canal treatment. Int Endod J.

[7] Mozayeni M.A., Hadian A., Bakhshaei P., Dianat O. (2015). Comparison of antifungal activity of 2% chlorhexidine, calcium hydroxide, and nanosilver gels against Candida albicans. J Dent.

[8] Javidi M., Zarei M., Afkhami F. (2011). Antibacterial effect of calcium hydroxide on intraluminal and intratubular Enterococcus faecalis. Iran Endod J.

[9] Vijayaraghavan R., Mathian V.M., Sundaram A.M., Karunakaran R., Vinodh S. (2012). Triple antibiotic paste in root canal therapy. J Pharm BioAllied Sci.

[10] Mozayeni M.A., Haeri A., Dianat O., Jafari A.R. (2014). Antimicrobial effects of four intracanal medicaments on enterococcus faecalis: an in vitro study. Iran Endod J.

[11] Dewi A., Upara C., Krongbaramee T., Louwakul P., Srisuwan T., Khemaleelakul S. (2021). Optimal antimicrobial concentration of mixed antibiotic pastes in eliminating *Enterococcus faecalis* from root dentin. Aust Endod J.

[12] Hegde V., Arora S. (2015). Effect of intracanal medicaments on push-out bond strength of Smart-Seal system. J Conserv Dent.

[13] Corrêa J.M., Mori M., Sanches H.L., Cruz ADd, Poiate E., Poiate I.A.V.P. (2015). Silver nanoparticles in dental biomaterials. Int J Biomater.

[14] Afkhami F., Elahy S., Mahmoudi-Nahavandi A. (2017). Spectrophotometric analysis of crown discoloration following the use of silver nanoparticles combined with calcium hydroxide as intracanal medicament. J Clin Exp Dent.

[15] Afkhami F., Pourhashemi S.J., Sadegh M., Salehi Y., Fard M.J.K. (2015). Antibiofilm efficacy of silver nanoparticles as a vehicle for calcium hydroxide medicament against *Enterococcus faecalis*. J Dent.

[16] Basrani B., Santos J.M., Tjäderhane L. (2002). Substantive antimicrobial activity in chlorhexidine-treated human root dentin. Oral Surg Oral Med Oral Pathol Oral Radiol Endod.

[17] Carrilho M.R., Carvalho R.M., Sousa E.N. (2010). Substantivity of chlorhexidine to human dentin. Dent Mater.

[18] Mohammadi Z., Shahriari S. (2008). Residual antibacterial activity of chlorhexidine and MTAD in human root dentin in vitro. J Oral Sci.

[19] Singh H., Kapoor P., Dhillon J., Kaur M. (2014). Evaluation of three different concentrations of Chlorhexidine for their substantivity to human dentin. Indian J Dent.

[20] Miles A.A., Misra S., Irwin J. (1938). The estimation of the bactericidal power of the blood. J Hyg.

[21] Bazvand L., Aminozarbian M.G., Farhad A., Noormohammadi H., Hasheminia S.M., Mobasherizadeh S. (2014). Antibacterial effect of triantibiotic mixture, chlorhexidine gel, and two natural materials Propolis and Aloe vera against *Enterococcus faecalis*: an ex vivo study. Dent Res J.

[22] Arias-Moliz M.T., Ferrer-Luque C.M., González-Rodríguez M.P., Navarro-Escobar E., de Freitas M.F., Baca P. (2012). Antimicrobial activity and *Enterococcus faecalis* biofilm formation on chlorhexidine varnishes. Med Oral Patol Oral Cir Bucal.

[23] Afkhami F., Karimi M., Bahador A., Ahmadi P., Pourhajibagher M., Chiniforush N. (2020). Evaluation of antimicrobial photodynamic therapy with toluidine blue against *Enterococcus faecalis*: laser vs LED. Photodiagnosis Photodyn Ther.

[24] Sedgley C., Lennan S., Appelbe O. (2005). Survival of *Enterococcus faecalis* in root canals ex vivo. Int Endod J.

[25] Adl A., Hamedi S., Motamedifar M., Sobhnamayan F. (2014). The ability of triple antibiotic paste and calcium hydroxide in disinfection of dentinal tubules. Iran Endod J.

[26] Asnaashari M., Ashraf H., Rahmati A., Amini N. (2017). A comparison between effect of photodynamic therapy by LED and calcium hydroxide therapy for root canal disinfection against Enterococcus faecalis: a randomized controlled trial. Photodiagnosis Photodyn Ther.

[27] Rosenthal S., Spångberg L., Safavi K. (2004). Chlorhexidine substantivity in root canal dentin. Oral Surg Oral Med Oral Pathol Oral Radiol Endod.

[28] Mahendra A., Koul M., Upadhyay V., Dwivedi R. (2014). Comparative evaluation of antimicrobial substantivity of different concentrations of chlorhexidine as a root canal irrigant: an *in vitro* study. J Oral Biol Craniofac Res.

[29] Ballal V., Kundabala M., Acharya S., Ballal M. (2007). Antimicrobial action of calcium hydroxide, chlorhexidine and their combination on endodontic pathogens. Aust Dent J.

[30] Mohammadi Z., Abbott P. (2009). On the local applications of antibiotics and antibiotic-based agents in endodontics and dental traumatology. Int Endod J.

[31] Sabrah A.H., Yassen G.H., Spolnik K.J., Hara A.T., Platt J.A., Gregory R.L. (2015). Evaluation of residual antibacterial effect of human radicular dentin treated with triple and double antibiotic pastes. J Endod.

[32] Althumairy R.I., Teixeira F.B., Diogenes A. (2014). Effect of dentin conditioning with intracanal medicaments on survival of stem cells of apical papilla. J Endod.

[33] Berkhoff J.A., Chen P.B., Teixeira F.B., Diogenes A. (2014). Evaluation of triple antibiotic paste removal by different irrigation procedures. J Endod.

[34] Hoshino E., Kurihara-Ando N., Sato I. (1996). In-vitro antibacterial susceptibility of bacteria taken from infected root dentine to a mixture of ciprofloxacin, metronidazole and minocycline. Int Endod J.

[35] Kishen A., Peters O.A., Zehnder M., Diogenes A.R., Nair M.K. (2016). Advances in endodontics: potential applications in clinical practice. J Conserv Dent.

[36] Javidi M., Afkhami F., Zarei M., Ghazvini K., Rajabi O. (2014). Efficacy of a combined nanoparticulate/calcium hydroxide root canal medication on elimination of Enterococcus faecalis. Aust Endod J.

[37] Sofi W., Gowri M., Shruthilaya M., Rayala S., Venkatraman G. (2012). Silver nanoparticles as an antibacterial agent for endodontic infections. BMC Infect Dis.

